# Higher maternal autonomy is associated with reduced child stunting in Malawi

**DOI:** 10.1038/s41598-021-83346-2

**Published:** 2021-02-16

**Authors:** Zizwani Brian Chilinda, Mark L. Wahlqvist, Meei-Shyuan Lee, Yi-Chen Huang

**Affiliations:** 1 Graduate Institute of Public Health, China Medical University, 91 Hsueh-Shih Road, North District, Taichung City, 40402 Taiwan; 2 Department of Nutrition, China Medical University, 91 Hsueh-Shih Road, North District, Taichung City, 40402 Taiwan; 3 School of Public Health, National Defense Medical Center, No. 161, Section 6, Minquan East Road, Neihu District, Taipei City, 11490 Taiwan; 4 Institute of Population Health Sciences, National Health Research Institutes, 35 Keyan Road, Zhunan, Miaoli County 35053 Taiwan; 5 Monash Asia Institute, Monash University, 5th Floor, H Building, 900 Dandenong Road, Caulfield East, VIC 3145 Australia

**Keywords:** Diseases, Health care, Health occupations, Medical research, Risk factors, Signs and symptoms

## Abstract

Child undernutrition is a major health problem in Malawi. We assessed the association between maternal autonomy and child stunting in Malawi. We utilized nationally representative pooled cross-sectional data from the 2010 and 2015/16 Malawi Demographic and Health Surveys (MDHS), which included 7348 mother (28.1 ± 6.8 years, range 15–49 years)—child (27.6 ± 16.7 months, range 0–59 months) pairs. Maternal autonomy composite scores captured decision-making power, tolerance of domestic violence, and financial independence. The nutritional outcome measure was stunting (height-for-age z score < – 2). Logistic regression assessed associations between maternal autonomy and stunting, and dominance analysis evaluated the relative importance of the associated factors. From the two surveys combined, 39.2% were stunted. Stunting decreased from 45.0% in 2010 to 34.6% in 2015/16; concurrently, maternal autonomy improved and was evidently associated with stunting (aOR = 0.81, 95% CI = 0.71, 0.93; *p* = 0.002). However, this association was probably mediated by other factors associated with improved child nutrition, including maternal education and family wealth, which, along with child age, were associated with stunting in the dominance analysis. Concurrent interventional programs may also have contributed to the decrease in stunting between the surveys, thus moderating the effect of maternal autonomy.

## Introduction

Globally, nearly 45% of all deaths of children younger than five years are attributable to undernutrition, with half of these deaths occurring in developing countries^1,2^. Child undernutrition impairs cell-mediated immunity, which increases the incidence and severity of diseases such as diarrhea, measles, tuberculosis, and malaria in early life^3^. Furthermore, undernutrition in the first two years of a child’s life is associated with poor health in later life, including cardio-metabolic diseases and impaired cognitive function with adverse educational and economic outcomes^4,5^.

In Malawi, 23% of under-five mortality is associated with undernutrition and an annual gross domestic product loss of 10.3%^6^. The 2015/16 Malawi Demographic and Health Survey (MDHS) revealed that more than one-third of under-five children were stunted^7,8^. This highlights the magnitude of child undernutrition in Malawi. Between 2010 and 2015/6, Malawi implemented a National Nutrition Policy and Strategic Plan (NNPSP)^9,10^ and was a participant in the international Scaling-Up Nutrition (SUN) initiative, which provided an opportunity to observe potential improvements in factors that might promote child nutrition^11,12^. However, despite these national nutrition initiatives and a subsequent ten-point reduction in the prevalence of child stunting between 2010 and 2015/16, the proportion of Malawian children who are stunted (37%) remains high^13,14^.

The United Nations Children’s Fund (UNICEF) conceptual framework categorizes the causes of child undernutrition as follows: (a) basic causes, which involve systemic-level challenges that reflect the structural and political processes in each society, including social, agricultural, food system, economic, environmental, and political issues; (b) underlying causes, which comprise insufficient household food security, inadequate care, poor feeding practices, lack of access to health services, and inhabitation of unhealthy environments where people lack access to safe water and may not practice hygienic waste disposal; and (c) immediate causes, which represent the impact of the basic and underlying causes at the individual level through inadequate dietary intake and diseases such as diarrhea, pneumonia, malaria, and measles^15^.

Maternal autonomy is among the resources required for optimal child care practices within the UNICEF conceptual care model^15,16^. A lack of maternal autonomy can contribute to child undernutrition, particularly in low-income countries. This autonomy depends on factors such as education, employment, household wealth, and status, which can be determinants of child nutrition and long-term life outcomes^17^.

While Malawi’s policies and programs prioritize food security and nutrition, they have not addressed the persistent gender gaps that impede the attainment of women empowerment^18^. Overall, Malawi ranked 116 out of 153 countries in the World Economic Forum Global Gender Gap Report 2020, with economic participation and opportunity (113/153) and educational attainment (128/153) being the two main gender gaps that the country faces^19^. Moreover, most women in Malawi lack control over land resources, even when they legally possess them. This is not withstanding the vital role that women play in producing food for their families. The lack of women’s control over such resources contributes to their disempowerment; it renders them vulnerable to poverty^20^, thus contributing to chronic child undernutrition^21^ and food insecurity^22^.

**Figure 1:**
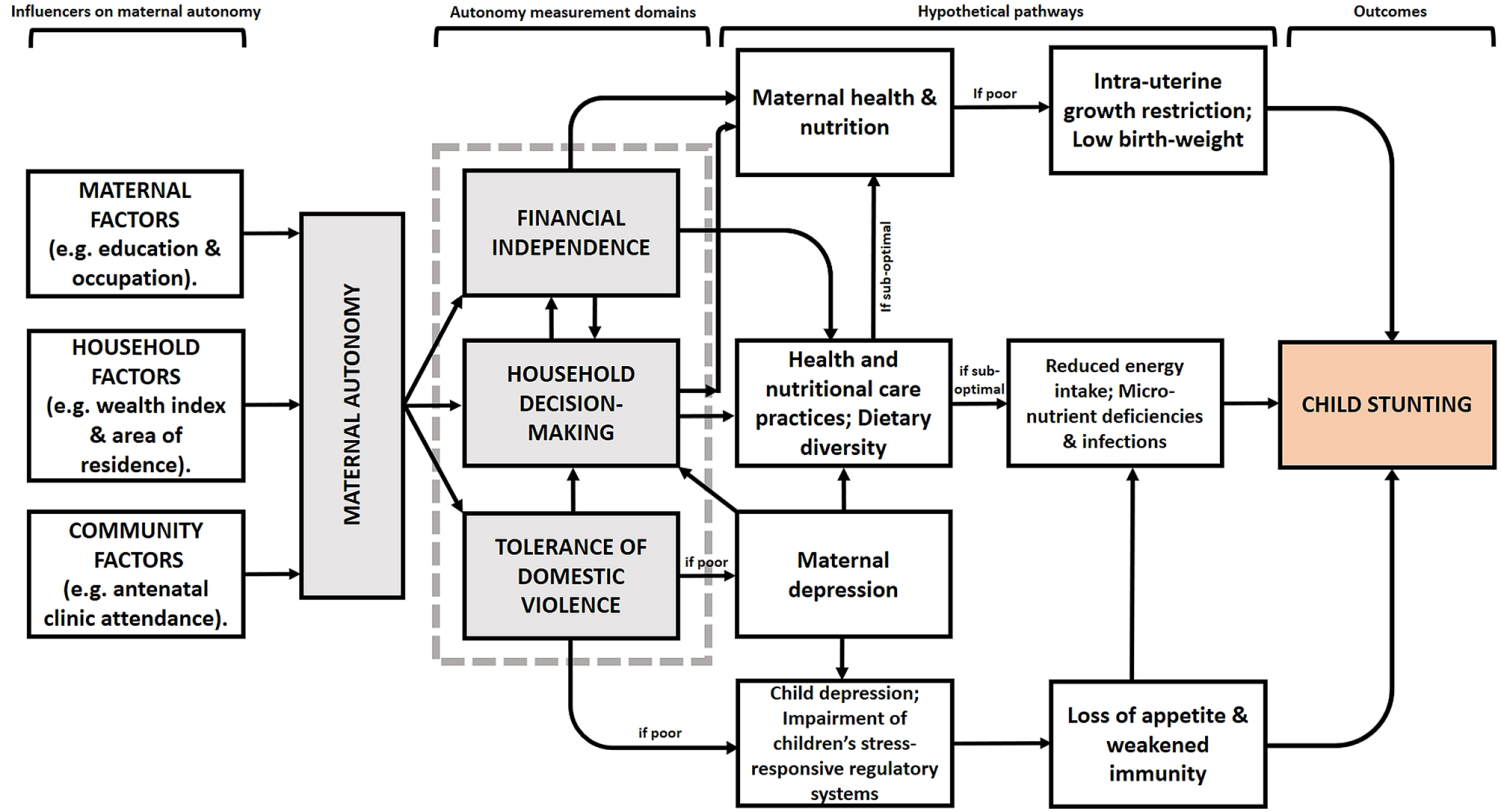
Conceptual framework for pathways linking maternal autonomy and child stunting.

On the basis of previous literature, we developed a conceptual framework (Fig. 1) that highlights the pathways through which maternal factors might influence child stunting in Malawi. We focused on maternal autonomy.

Maternal autonomy comprises three domains: household decision-making power, tolerance of domestic violence, and financial independence. Our framework was based on the concept that maternal autonomy is influenced by a combination of individual, household, and community characteristics^23^. Because mothers are often the primary caregivers of young children, they have considerable control over factors critical for a child’s well-being, including food preparation and storage, feeding practices, psychosocial care, hygiene and health practices, and newborn care^24^. Women with high decision-making power channel household resources toward improving their caring practices, thus improving the health and nutritional status of their children^17^.

Increasing women’s financial independence increases their bargaining power in the household and thus expands their autonomy^25^. Assets that women have at their disposal constitute a source of their financial independence that improves their security. Accordingly, land and/or house ownership should be a source of empowerment in that it increases women’s security and influence and enhances their control over household decisions^26^. A survey of five Asian countries revealed that women who possessed land in India and Thailand had higher domestic financial independence than those who did not^27^.

A mother’s exposure to domestic violence may trigger behavioral (smoking, alcohol, and drug use), psychological (depression, anxiety, reduced self-esteem, and overall poor mental health), and physical (injury, disability, and fatigue) risks as well as nutritional disorders (anemia and inappropriate weight gain)^28^. Maternal risks may negatively affect a child’s early growth and nutritional status. Such risks may exert their effects through the following pathways: maternal, antenatal, delivery and postnatal care, as well as infant and child care^29^. Maternal exposure to domestic violence may dysregulate a child’s stress response system as well as impair energy metabolism, immunity, mental and cognitive function, reproduction, and growth^30^.

We conducted this study with the aim of assessing the association between maternal autonomy and stunting in under-five children in Malawi by using data from the MDHS. We hypothesized that maternal autonomy is inversely associated with child stunting (height-for-age). Moreover, we hypothesized that concurrent health and nutrition programs in Malawi benefit both maternal autonomy and child stunting and their association.

## Methods

### Population and study design

The target population comprised reproductive-aged Malawian women with children. This study applied a cross-sectional design and used data obtained from the 2010 and 2015/16 MDHSs. Information was collected through face-to-face interviews conducted with women aged 15–49 years who had children under the age of 5 years.

The MDHSs applied the sampling frame that was used for the 2008 Malawi Population and Housing Census. Both MDHSs involved a stratified two-stage probability sampling design in three regions (northern, central, and southern), and their data constituted a nationally representative sample. In the first stage, 849 standard enumeration areas (SEAs; 158 urban and 691 rural areas) were selected for the 2010 MDHS, and 850 SEAs (173 urban and 677 rural areas) were selected for the 2015/16 MDHS; probability was proportional to the SEA size, and the selection process was unique for each sampling stratum. In the second stage, through an equal probability systematic selection method, 20 households per urban cluster and 35 per rural cluster were selected for the 2010 MDHS, and 30 households per urban cluster and 33 per rural cluster were selected for the 2015/16 MDHS^13,14^.

### Participant selection

**Figure 2:**
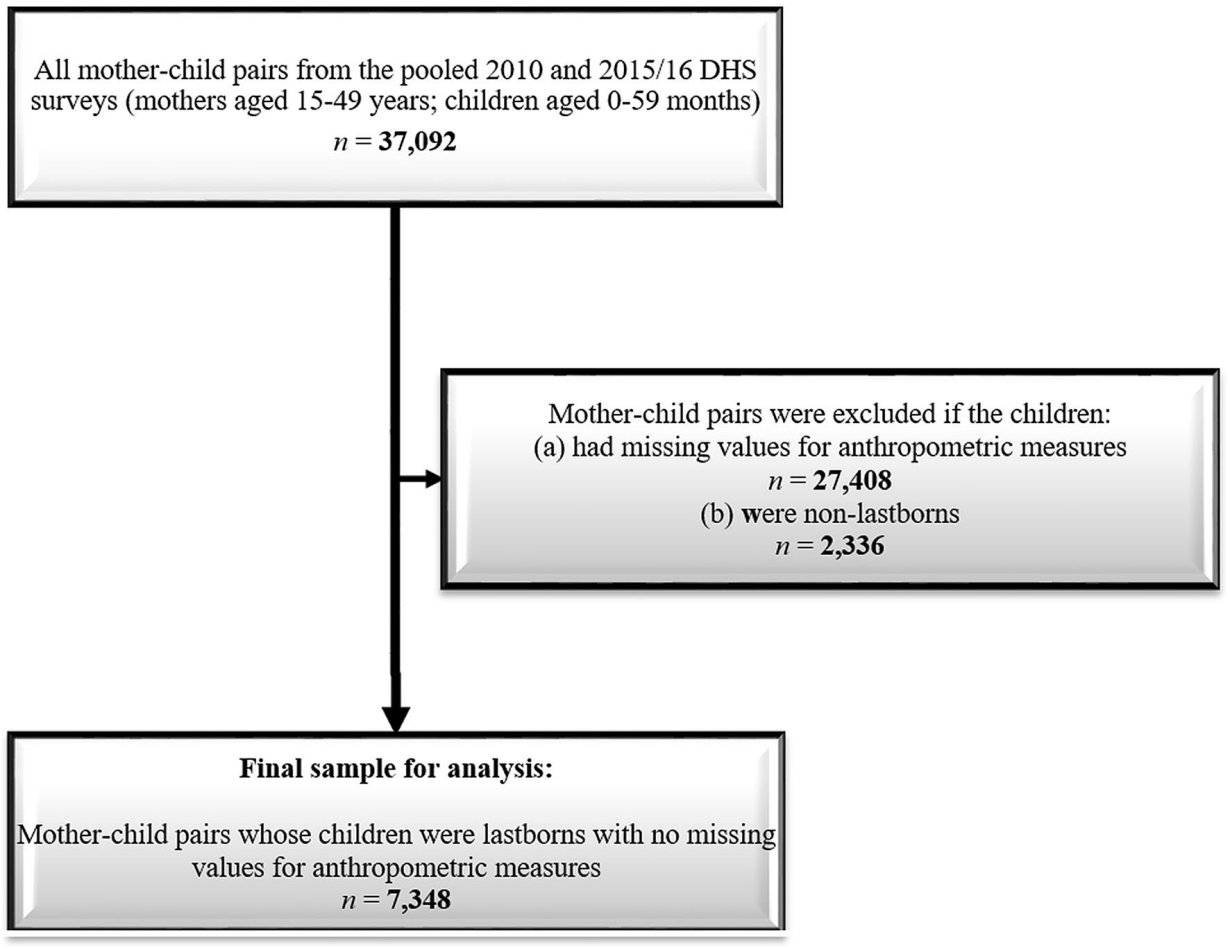
Participant selection flowchart.

The pooled 2010 and 2015/16 MDHS data comprised 37,092 mother–child pairs. We excluded 27,408 children for having incomplete height, weight, or age data. Furthermore, we restricted our analysis to mother–child pairs in which the lastborn was the index child; consequently, we excluded an additional 2,336 pairs in which the children were not lastborns. Our final analysis sample contained 7,348 mother–child pairs (Fig. 2).

### Dependent variable

#### Child anthropometry

Weight was measured using UNICEF-approved scales, and height was measured using measuring boards specifically designed for survey settings by Shorr Productions. Recumbent length was measured for children aged ≤ 24 months old, and standing height was measured for children aged 25–59 months. A child’s nutritional status was determined by their height-for-age z-score (HAZ) according to the WHO 2006 growth reference standards^31^. Children with HAZs below − 2 standard deviations (SD) from the median of the reference population were classified as stunted^32^.

### Independent variable

#### Maternal autonomy

Maternal autonomy was assessed using variables in the following three domains: (a) household decision-making power, (b) tolerance of domestic violence, and (c) financial independence^17^. These variables and their subdomains have been extensively used across several African countries and validated through confirmatory factor analysis in multi-country studies using questionnaires^33^. The original questionnaires were in English and were translated into the local Chichewa and Tumbuka languages by highly skilled and experienced survey specialists. They were then back-translated into English and pretested in training settings. Data were collected by trained enumerators^14,34^.

Decision-making power was defined as a woman’s ability to have the final say about (a) her own health care, (b) making large household purchases, and (c) visits to family or relatives. These variables were dichotomized into the following groups: “has no say” (0), if one or more persons other than the interviewed mother makes the decision; and “has say” (1), if the respondent has part (with spouse/partner or another person) or sole decision-making power. Table 1 lists the detailed scoring criteria.

**Table 1:** Scoring of the three indicators used in the development of the maternal autonomy index.

Domains and conditions	Scoring criteria
**A woman’s involvement in household decision-making. Includes 3 decisions—final say on**	Participates in all 3 decisions = 2 Participates in 1 or 2 decisions = 1 Does not participate in any decisions = 0
• Her own healthcare	
• Making large household purchases, and	
• Making visits to family and relatives)	
**A woman’s tolerance of domestic violence. Includes whether or not a woman thinks it is justifiable for her husband to beat her in the following 5 situations**	Thinks it is not justifiable in any situation = 4 Thinks it is justifiable in 1 or 2 situations = 3 Thinks it is justifiable in 3 situations = 2 Thinks it is justifiable in 4 situations = 1 Thinks it is justifiable in all the 5 situations = 0
• When she goes out without telling her husband	
• When she neglects the children	
• When she argues with her husband,	
• When she denies her husband sex and	
• When she burns the food	
**A woman’s financial independence. Includes ownership of either**	Owns a house, land, or both alone or jointly with husband = 1 Does not own any house, land = 0
• House, or	
• Land	

The range of maternal autonomy scores was 0–7, with 0–3 indicating low levels of maternal autonomy; 4–5 indicating moderate levels of maternal autonomy; and 6–7 indicating high levels of maternal autonomy.

A woman’s tolerance of domestic violence was assessed according to their responses to five questions. Respondents were asked to indicate whether wife beating is justified under the following circumstances: (a) goes out without her husband, (b) neglects the children, (c) argues with her husband, (d) refuses to have sex with her husband, or (e) burns the food (Table 1 for scoring criteria).

A woman’s financial independence was assessed according to her ownership of (a) a house or (b) land (Table 1 for scoring criteria). The scores of the three domains were summed to derive the maternal autonomy index (ranging from 0 to 7). This index is also referred to as the women empowerment index (WEI)^2^. Women were categorized into three groups according to their WEI scores: low autonomy (0–3), moderate autonomy (4–5), and high autonomy (6–7)^35^.

### Covariates

We categorized our covariates into four levels: child, maternal, household, and community factors^17^.

Child factors included age (in months; 0–11, 12–23, 24–35, 36–47, or 48–59 months), sex (boy or girl), birth order (1, 2, 3, or 4+), and whether the child had diarrhea recently (yes or no).

Maternal factors included age (in years; 15–19, 20–24, 25–29, 30–34, 35–39, 40–44, or 45–49), marital status (not married, married/cohabiting, or divorced/separated/widowed), number of wives other than mother (0, 1, or 2+), body mass index (BMI; underweight: < 18.5 kg/m^2^; normal: 18.5–24.9 kg/m^2^; or overweight: ≥ 25.0 kg/m^2^)^36^, education level (no education, primary, secondary or higher), occupation (housekeeping or agricultural, professional, service, or manual labor), breastfeeding duration (never breastfed, stopped breastfeeding, or still breastfeeding), and current pregnancy status (pregnant or not pregnant).

Household factors included household wealth index, type of place of residence (urban or rural), number of household (HH) members (1–5, 6–10, or 11+), number of children under 5 years (1, 2, or 3 +), and region (northern, central, or southern). The household wealth index was a composite measure of a household’s cumulative living standard and was derived according to a household’s ownership of selected assets, such as televisions and bicycles, materials used for constructing the house, access to safe water, sanitation facilities, and other household characteristics. Household asset scores were derived using a principal component analysis; thus, households were classified into quintiles (poorest, poorer, middle, richer, and richest) according to weighted household asset scores^37^.

Community factors included the family planning method currently used (none, traditional/folkloric, or modern), number of antenatal care (ANC) visits during the previous pregnancy (never attended, < 4 visits [inadequate], or ≥ 4 visits [recommended])^38^, whether the mother received iron and folic acid (IFA) supplementation during the previous pregnancy (yes or no), and whether the mother received anthelminthic drugs during previous pregnancy (yes or no).

### Statistical analysis

We performed all statistical analyses using SPSS Version 21.0 (IBM, Armonk, NY, USA)^39^ and STATA version 14.1 (StataCorp, College Station, TX, USA)^40^. The STATA survey (svy) command was used to analyze the MDHS design effect. Participant characteristics are reported as weighted frequencies and percentages. Pearson’s chi-square test was performed to assess the differences in the distribution of independent variables between groups (child stunting [Yes/No]) and to check for possible confounding among the covariates (see Supplementary Table S1). Variables with a *p*-value of < 0.05 were regarded as statistically significant. All independent variables that were significant (*p* < 0.05) in the univariable analysis were tested for multicollinearity using the variance inflation factor (VIF)^41^ before being included in the final logistic regression analyses. No collinearity was observed among the independent variables because all VIF values were < 10^42^ (mean VIF value: 1.31).

The generalized linear mixed model (GLMM) was used to estimate the effects of independent variables on the outcome after all covariates were controlled for. Because of the hierarchical structure of the MDHS data, the SEA and survey year (2010 and 2015/16) variables were used to adjust for any possible correlation in the responses of individuals (i.e., mother–child pairs) from the same clusters^43^. Survey year represents potential secular change or trend, relevant to all variables under consideration. Thus, unlike other covariates, multiple variables are associated with it. Therefore, it must be considered as common to all variables of interest over the study time frame, or a contextual description of events during this time frame be generated as an explanatory narrative. We applied a sampling weight in the GLMM. Furthermore, we explored possible interaction effects between the covariates and maternal autonomy on child stunting (see Supplementary Table S2). Crude (ORs) and adjusted odds ratios (aORs) and their corresponding 95% confidence intervals (CIs) were derived to quantify the strength of the association between our dependent and independent variables in the regression analysis without (ORs) and with the influence of the covariates on the association (aORs). *K*-fold cross-validation was performed to estimate the accuracy of the predictive model in practice (see Supplementary Table S3 and Supplementary Fig. S1).

We subsequently conducted dominance analysis to determine the relative importance of the independent variables with respect to child stunting; we used the McFadden R^2^ statistic to estimate the variance explained^44^. Relative importance refers to the proportionate contribution each independent variable makes to R^2^ if we consider both its individual correlation with the outcome variable and its effect when combined with other independent variables in the regression equation^45^. The general dominance weight of each independent variable was divided by the combined general dominance weight of all independent variables and multiplied by 100% to yield a normed measure representing the proportionate contribution made by each independent variable to the total variance. Dominance analysis accounts for an individual variable’s direct effect, total effect (conditional on all other independent variables), and partial effect (conditional on subsets of independent variables) in terms of its contribution to the total variance^46^.

### Ethical considerations

The 2010 and 2015/16 MDHSs were conducted by the National Statistics Office and the Community Health Sciences Unit, Malawi. The survey protocol was reviewed and approved by the Institutional Review Board of ICF Macro and the Malawi Health Sciences Research Committee. The datasets are publicly available at http://dhsprogram.com/data/available-datasets.cfm upon approval of a written request.

## Results

### Child stunting and maternal autonomy situation and changes in Malawi

#### Child stunting

Among the 7,348 under-five children selected in this study, 39.2% were stunted. The weighted prevalence of stunting changed favorably from 45.0 to 34.6% from 2010 to 2015/16, respectively. The prevalence of stunting in the pooled original (full) data was 41.5%; the prevalence in these data changed favorably from 47.0 to 36.6% from 2010 to 2015/16, respectively. These results thus demonstrate that no significant differences in the prevalence of stunting existed between our study subset and the original MDHS sample.

For the pooled data we selected (i.e., subset), the mean age of the children was 27.6 months [standard deviation (SD): 16.7]. The children’s sex ratios were similar, and most children had no recent diarrheal disease (see Supplementary Table S4).

#### Maternal autonomy and other maternal factors

Most women in our pooled sample had moderate levels of autonomy (48.9%), which was comparable to the proportion (52.1%) observed in the full survey data. The maternal autonomy index was higher in 2015/16 (37.8%) than in 2010 (17.3%) (see Supplementary Table S4). The prevalence of child stunting decreased as maternal autonomy increased. Low maternal autonomy was associated with a stunting prevalence of 42.5%, and high maternal autonomy was associated with a prevalence of 36.5% (*p* < 0.001). Moreover, significantly more women with high maternal autonomy had attained a secondary or higher education (25.3% vs. 12.6%), were working in occupations with remunerative potential (33.9% vs. 23.4%), had only one under-five-year-old child (58.4% vs. 51.7%), and were dewormed antenatally (47.5% vs. 41.5%) compared to women with low or moderate autonomy (*p* < 0.001, respectively) (Table 2).

**Table 2:** Characteristics of pooled participants by maternal autonomy (*N* = 7,348).

Characteristics	Low autonomy (*n* = 1643)	Moderate autonomy (*n* = 3596)	High autonomy (*n* = 2109)	*p value*
	%	%	%	
Child stunting	42.5	39.3	36.5	**< 0.001**
**Child factors**				
Age (months)				**< 0.001**
0–11	24.3	26.3	25.1	
12–23	31.6	27.3	23.5	
24–35	20.0	22.0	22.9	
36–47	15.3	15.3	17.6	
48–59	8.8	9.1	10.8	
Age (months) Mean (SD)	15.1	15.2	18.8	
Sex, Girl	50.1	51.8	50.9	0.737
Child had diarrhea recently, Yes	24.2	22.5	19.7	**0.009**
**Maternal factors**				
Age (years)				**< 0.001**
15–19	7.5	9.4	4.8	
20–24	29.0	28.6	25.0	
25–29	23.9	28.2	26.4	
30–34	18.3	16.6	22.0	
35–39	12.3	10.8	14.4	
40–44	6.6	4.2	5.4	
45–49	2.4	2.2	2.0	
Age (years) Mean (SD)	6.9	7.0	6.7	
Marital status				**< 0.001**
Not married	2.0	4.7	0.0	
Married/cohabiting	92.7	79.1	100	
Divorced/separated/widowed	5.3	16.2	0.0	
Number of other wives^††^				**0.002**
0	86.2	88.2	89.2	
1	12.5	10.7	10.2	
2 +	1.3	1.1	0.6	
Mother’s BMI (kg/m^2^)^††^				**< 0.001**
Underweight (< 18.5)	5.8	6.1	4.6	
Normal (18.5–24.9)	76.5	76.6	70.0	
Overweight (≥ 25.0)	17.7	17.3	25.4	
Mother’s education level				**< 0.001**
No education	15.5	14.8	11.4	
Primary	71.9	65.6	63.3	
Secondary or higher	12.6	19.6	25.3	
Occupation				**< 0.001**
Housekeeping/agriculture	76.6	73.4	66.1	
Professional/service/manual labor	23.4	26.6	33.9	
Breastfeeding duration^††^				**< 0.001**
Never breastfed	1.2	1.2	1.4	
Stopped breastfeeding	42.8	44.5	50.7	
Still breastfeeding	56.0	54.3	47.9	
Current pregnancy status, Pregnant	9.0	8.7	8.4	0.234
**Household Factors**				
Family wealth index				**< 0.001**
Poorest	21.6	20.4	18.5	
Poorer	25.1	21.3	22.3	
Middle	23.6	19.6	18.1	
Richer	19.5	18.3	19.1	
Richest	10.2	20.4	22.0	
Number of HH members				**< 0.001**
1–5	55.5	53.9	55.5	
6–10	42.6	42.8	43.0	
11 +	1.9	3.3	1.5	
Number of children under the age of 5^††^				**< 0.001**
1	51.7	50.8	58.4	
2	40.3	40.5	35.2	
3 +	8.0	8.7	6.4	
Type of area of residence, Rural	93.6	82.6	82.0	**< 0.001**
Region				**< 0.001**
Northern	14.8	8.7	11.7	
Central	42.4	45.0	45.1	
Southern	42.8	46.3	43.2	
**Community Factors** **(Community Health Services)**				
Antenatal attendence				0.084
Never attended	1.5	1.2	1.9	
< 4 visits (inadequate)	50.6	49.7	46.6	
≥ 4 visits (recommended)	47.9	49.1	51.5	
IFA supplementation, Yes	90.3	89.9	90.6	0.755
Received anthelmintic drugs during previous pregnancy, Yes	41.5	38.8	47.5	**< 0.001**

*p*, Pearson’s chi-square *p* value; ^††^Total count < *n*; BMI, body mass index; IFA, iron and folic acid; SD, standard deviation. Statistical significance was set at *p* < 0.05. Significant *p* values are in boldface.

### Autonomy and child nutritional status

Table 3 shows the mediating effects of the covariates on the association between maternal autonomy and child stunting. Mothers with high maternal autonomy were less likely to have stunted children (OR = 0.84, 95% CI = 0.73, 0.96; *p* = 0.011; Model 1). After adjustment for child age and sex, we observed that the association between maternal autonomy and child stunting was moderately attenuated by the unfavorable effect of older age and favorable effect of the female sex on stunting (aOR = 0.85, 95% CI = 0.74, 0.97; *p* = 0.020; Model 2).

**Table 3:** Sociodemographic factors mediating the effect of maternal autonomy on child stunting.

Model	Covariates	OR (95% confidence interval)				*p* value
		Maternal autonomy				
		Low (ref)	Moderate	*p* value	High	
1	None^φ^	1.00	0.89 (0.79, 1.01)	0.072	**0.84 (0.73, 0.96)**	**0.011**
2	Child’s age + sex^φ^	1.00	0.91 (0.80, 1.03)	0.116	**0.85 (0.74, 0.97)**	**0.020**
3	Child’s age + sex + mother’s age^φ^	1.00	0.90 (0.79, 1.02)	0.098	**0.86 (0.75, 0.99)**	**0.031**
4	Child’s age + sex + mother’s BMI^φ^	1.00	0.90 (0.79, 1.02)	0.096	**0.87 (0.75, 1.00)**	**0.043**
5	Child’s age + sex + mother’s education level^φ^	1.00	0.92 (0.81, 1.04)	0.199	0.89 (0.77, 1.02)	0.099
6	Child’s age + sex + current pregnancy status^φ^	1.00	0.92 (0.80, 1.03)	0.120	**0.85 (0.74, 0.98)**	**0.021**
7	Child’s age + sex + family wealth index^φ^	1.00	0.93 (0.83, 1.06)	0.289	0.91 (0.79, 1.05)	0.193
8	Child’s age + sex + area of residence^φ^	1.00	0.93 (0.83, 1.06)	0.286	0.89 (0.77, 1.03)	0.103
9	Child’s age + sex + IFA^φ^	1.00	0.90 (0.80, 1.02)	0.110	**0.85 (0.74, 0.97)**	**0.020**
10	Child’s age + sex + anthelmintic drugs^φ^	1.00	0.91 (0.80, 1.03)	0.116	**0.85 (0.74, 0.97)**	**0.020**
11	All covariates mentioned above^φ^	1.00	0.93 (0.82, 1.05)	0.226	0.94 (0.82, 1.09)	0.396
12	All covariates mentioned above except mother’s education level, family wealth index, and area of residence^φ^	1.00	0.90 (0.79, 1.02)	0.087	0.87 (0.76, 1.01)	0.059
13	All covariates mentioned above except mother’s education level, family wealth index, and area of residence^‡^	1.00	0.94 (0.83, 1.07)	0.343	**0.81 (0.71, 0.93)**	**0.002**

^φ^Adjusted for multilevel clustering using the *standard enumeration area (SEA)* and *survey year* (MDHS 2010–2015/16) variables. ^‡^Adjusted for clustering using the *SEA* variable only. OR, odds ratio; BMI, body mass index; IFA, iron and folic acid supplementation; MDHS, Malawi Demographic and Health Survey; ref, reference category. Statistical significance was set at *p* < 0.05. Significant *p* values are in boldface.

We still observed moderate increases in the ORs in the subsequent models after further adjustment for maternal age, maternal BMI, current pregnancy, IFA supplementation, and receipt of anthelmintic drugs in the previous pregnancy; this finding suggests that these covariates are partially independent of maternal autonomy with respect to their effects on child stunting. Nonetheless, the probability of stunting remained low and significant, indicating a negative association between maternal autonomy and child stunting.

The effect of maternal autonomy was no longer significant in the models adjusted for the mother’s education level (*p* = 0.099; Model 5), family wealth index (*p* = 0.193; Model 7), and area of residence (*p* = 0.103; Model 8). Finally, after we included all covariates in the model, we observed no further change in the effect of maternal autonomy on child stunting (aOR = 0.94, 95% CI = 0.82, 1.09; *p* = 0.396; Model 11). The effect of maternal autonomy on child stunting remained non-significant after the exclusion of the mother’s education level, family wealth index, and area of residence (*p* = 0.059; Model 12).

Finally, along with maternal education level, family wealth index, and area of residence, we excluded survey year as one of the control variables for clustering to explore whether it had any effect on the association between maternal autonomy and child stunting. We noted a significant inverse association between our predictor and outcome variables, with the corresponding model having the lowest aOR among all models (aOR = 0.81, 95% CI = 0.71, 0.93; *p* = 0.002; Model 13).

### Relative importance of the factors associated with child stunting

Table 4 presents the dominance analysis results. Child age (in months), family wealth index, and maternal education level were the top three variables among the 12 that we evaluated, including maternal autonomy, and accounted for 55% of the total variance (22%, 15%, and 18%, respectively). Maternal education level ranked third, independent of autonomy. The maternal autonomy index was among the lowest-ranked covariates in the model.

**Table 4:** Relative importance of the factors stunting among children aged 0–59 months in Malawi by dominance analysis (*N* = 7348).

Variables	Child stunting		
	Dominance statistic	Standardized weight^††^	Rank
Maternal autonomy index	0.0012	0.03	9
Child’s age (months)	0.0093	0.22	1
Sex	0.0036	0.09	5
Mother’s age (years)	0.0003	0.01	11
Mother’s BMI	0.0060	0.14	4
Mother’s education level	0.0063	0.15	3
Breastfeeding duration	0.0021	0.05	7
Current pregnancy status	0.0016	0.04	8
Family wealth index	0.0075	0.18	2
Type of area of residence	0.0027	0.06	6
IFA supplementation	0.0003	0.01	12
Received anthelmintic drugs during previous pregnancy	0.0010	0.02	10
Overall Fit Statistic	0.0420		

^††^Standardized weight is the general dominance weight from McFadden R^2^ normed or standardized to be out of 100%. BMI, body mass index; IFA, iron and folic acid. Variables included in this dominance analysis are those fitted into the final model of the regression analysis.

## Discussion

This study revealed that higher maternal autonomy was associated with a lower risk of early childhood stunting in Malawi; these findings are consistent with those of studies conducted in Egypt^47^, Kenya^48^, and Tanzania^17^. Furthermore, maternal education level, family wealth index, area of residence, and survey year have probably moderated the association between maternal autonomy and childhood stunting, although their individual impact could not be clearly established because these determinants were highly correlated.

We considered survey year as a potential proxy variable for the presence of a national nutrition program and its associated initiatives which, according to our study design, entailed pooling the data of the two surveys with a 5-year gap (MDHS 2010 and MDHS 2015/16) to identify trends in any maternal autonomy–child stunting association.

Standardized weight estimates derived from our dominance analysis indicated the relative importance of putative associated factors for child stunting (Table 4). The top three associated factors—namely child age, family wealth index, and maternal education level—accounted for 55% of the variance. These findings strengthen our argument about the mediating role of maternal education and family wealth in the association between maternal autonomy and child stunting. This study revealed that the maternal autonomy index did not rank highly as an associated factor for child stunting.

Child age was associated with child stunting, with the likelihood of stunting increasing with age. Studies in Ethiopia have shown similar findings^49^. This may be because as children grow older, their energy and other nutritional needs increase^50^. Most children in our study resided in rural areas of Malawi, where poverty is rampant and most families have little to no access to nutritious diets. Stunting is a manifestation of chronic undernutrition^51^. Nevertheless, child stunting was less prevalent in the second survey, which was approximately 5 years after the first survey. The health relevance of **“**stature**”** is often unclear in studies on the association between maternal factors and child stunting. In developing economies, shortness is most likely caused by poor food availability, low food quality, inadequate food intake, recurrent infections, and psychosocial deprivation, leading to stunting, which may also be referred to as “unhealthy shortness.” Linking candidate determinants of child stunting is particularly difficult because stature provides an adaptive mechanism for overcoming nutritional disadvantages, sparing at least some of the possible adverse consequences of poor diets. Shorter people with less lean body mass have less nutritional requirements and are more suited to some socioecological settings (e.g., social infrastructure, latitude, altitude, topography)^52^. In the present study, ultimate growth velocity or stature is likely to have reflected trade-offs between various intrinsically-determined biomedical, and extrinsically-determined environmental and psychosocial factors. Given their multiplicity, the likelihood of any one factor accounting for shortness in a complex setting as in the present study is low.

We found that maternal education is a critical factor influencing maternal autonomy and childhood stunting. The inclusion of maternal education level in our regression model obscured the effect of maternal autonomy on child stunting. The most commonly used proxy measure of women's autonomy in earlier studies has been education^53^. Education has been identified as a critical enabler of women’s empowerment as it enhances their ability to make important decisions. For example, health outcomes of educated women and their children tend to be better as they are able to demand and access healthcare services^54^. Additionally, educated women have more knowledge than their uneducated counterparts; they are more employable and likely to be financially independent. All the aforementioned have been known to boost the status of women and their confidence in making decisions that naturally impact their health and that of their children^55^. Therefore, policies that are geared towards educating women could improve nutritional and health outcomes of their children.

Our study revealed that the odds of child stunting exhibited a downward trend as the wealth quintiles increased, indicating a relationship between household wealth and child nutrition. This finding is supported by those reported by Nepalese^56^ and Ethiopian^57^ studies. A possible explanation for this finding is that children from wealthier households might be fed diverse and more nutritious foods compared with those from poorer households. Our dominance analysis revealed that among the 13 variables analyzed, the household wealth index was the second most critical factor associated with child stunting. Hence, we recommend the enhancement of programs that integrate interventions geared toward improving the socioeconomic status of the poorest households in Malawi, such as the Malawi Social Cash Transfer Program (MSCTP); this is because such programs can improve children's dietary diversity and possibly their nutritional status^58^. Currently, the MSCTP targets only 10% of the poorest, labor-constrained households in each of Malawi’s 28 districts, typically those headed by elderly people who look after primary- or secondary-school-going orphans^59^. With nearly 70% of people living below the international poverty line in the country^60^, the 10% cap per district should be removed. In addition, the program should extend its beneficiary profile to include households with under-five children; this is because such children constitute one of the most vulnerable groups in society with considerable potential to experience positive changes in nutritional status.

Survey year represented the impact of interventions in the concurrent national nutrition programs that were operative before, during, or after the 2010–2015/16 period. One of such programs would have been the SUN project. Kanyuka et al. highlighted that since the inception of the SUN project in 2011, Malawi has scaled up direct nutrition interventions aimed at reducing childhood illnesses that inhibit the utilization of nutrients. Nutrition programs include the expansion of vitamin A supplementation and deworming for children, behavioral change interventions to promote proper nutrition during pregnancy, and adequate infant and young child feeding practices^61^. In addition, Malawi has embarked on nationwide implementation of community-based treatment campaigns for children with severe acute malnutrition through the community management of acute malnutrition (CMAM) approach, which is the core component of Malawi’s NNPSP^62^. This approach achieved 90% coverage across the country with donor support from development partners; we deduce that this coverage rate may have been the reason for much of the amelioration of the prevalence of child undernutrition between 2010 and 2015/16^8,63^. Nationally, more than 90% of children with severe acute malnutrition admitted to the CMAM outpatient therapeutic program between 2012 and 2015 were successfully treated, with < 2% deaths and < 7% defaults^62^; thus, this program reduced child stunting in Malawi by preventing repetitive cycles of acute malnutrition. These interventions are the major contributors to the national success in the fight against childhood undernutrition^61^. The impact of these interventions is thus likely to have moderated the association between maternal autonomy and child nutritional trend.

The present study extends the findings of a previous international study that was conducted by Desai and Johnson to examine the impact of women’s decision-making autonomy on child height-for-age, immunization, and survival in 12 countries, including Malawi; they reported that community support was a crucial factor^64^. The present study considered the maternal autonomy index as a composite variable and applied dominance analysis to regression models to ascertain maternal autonomy’s association with and relative importance in Malawian child undernutrition. Our findings provide insights into and hypotheses related to policy, societal, and household factors that might explain the associations reported herein and in the literature but required confirmation in Malawi. Our study findings elucidate the putative contributory factors to child stunting by using a large sample size; quality control measures; and contextual variables adjusted at the child, maternal, household, and community levels.

Our study also has some limitations. The cross-sectional design of our study precluded causative conclusions. Although designed to be nationally representative, the final sample comprised only 20% of the original sample considering missing data; thus, selection bias cannot be excluded. The study utilized secondary datasets, which, although large, lacked variables that could have resolved some outstanding questions, such as indicators of family planning, maternal time and resource constraints, and men’s involvement in childcare and feeding. More information about the mothers’ and children’s diets (with children’s diets stratified by age and growth) would have allowed the differentiation of healthy shortness from stunting. The datasets included household dietary intake only for children aged 6–23 months in the 2015–16 survey; they revealed that dietary diversity is positively associated with a more successful dietary transition from breastfeeding^65^. This conceivably provides a pathway for maternal autonomy to affect child nutrition (Fig. 1). Another limitation is that the MDHS data were based on a questionnaire and prone to fallible memory. Moreover, the data were collected from women only and did not include men’s viewpoints, which might confirm or repudiate the claims made by women. Ghuman et al. observed that maternal autonomy measurements change depending on whether the woman or her husband was interviewed^66^. Future studies can be modified accordingly.

In conclusion, maternal autonomy in Malawi improved from 2010 to 2015/16, and it was a determinant of child stunting. However, its effect was attenuated by other factors, such as maternal education and family wealth, which ranked among the top-three factors by dominance analysis. Thus, increasing overall maternal schooling in adulthood and expanding the national social cash transfer program for low-income families have potential to impact women empowerment and child stunting. Concurrent national nutrition programs may also have contributed to the substantial decrease in child stunting between surveys. In order to scale up community nutrition interventions aimed at improving child nutrition, there is need to integrate the national Early Childhood Development (ECD) program into the NNPSP and enhance community capacity. This would help deliver community nutrition activities through, for example, community-based childcare centers (CBCCs), care group volunteers (CGVs), and other existing platforms. Integration of the ECD program into the NNPSP would help increase community capacity and provide nutritious foods to CBCCs. The CGVs could reach a larger proportion of the population and impact both dietary diversity and child linear growth. Building capacity in this way with government and development partners would scale up nutrition and gender-sensitive activities, so empowering women and improving child nutrition. Our study demonstrates that women have the capacity to improve their autonomy as child nutrition improves in the sociocultural settings found in Malawi. Concurrently, women have aspirations related to their household and community well-being and prosperity as they pursue remunerative occupations. Thus, the competitive priorities that mothers faced in the present study have a policy bearing on nutrition and health programs for child development in Malawi.

## Supplementary Information

Supplementary Information
