# Supplementary material for: Higher maternal autonomy is associated with reduced child stunting in Malawi
Source: Sci Rep. 2021 Feb 16;11:3882. doi: 10.1038/s41598-021-83346-2 (PMC7886910; doi:10.1038/s41598-021-83346-2)
Supplement: Supplementary file 1 — Supplementary Information [file 41598_2021_83346_MOESM1_ESM.pdf]

# Higher maternal autonomy is associated with reduced child stunting in Malawi

**Zizwani Brian Chilinda**[1], **Mark L. Wahlqvist**[2,3,4,5], **Meei-Shyuan Lee**[3], **and Yi-Chen Huang** [2,*]

1 Graduate Institute of Public Health, China Medical University, 91 Hsueh-Shih Road, North District, Taichung City 40402, Taiwan
2 Department of Nutrition, China Medical University, 91 Hsueh-Shih Road, North District, Taichung City 40402, Taiwan
3 School of Public Health, National Defense Medical Center, No.161, Section 6, Minquan East Road, Neihu District, Taipei City 11490, Taiwan
4 Institute of Population Health Sciences, National Health Research Institutes, 35 Keyan Road, Zhunan, Miaoli County 35053, Taiwan
5 Monash Asia Institute, Monash University, 5th Floor, H Building, 900 Dandenong Road, Caulfield East, Victoria 3145, Australia
* yichenhuang@mail.cmu.edu.tw

| Characteristics | Child stunting | | p |
|---|---|---|---|
| | No (*n*=4467) | Yes (*n*=2881) | |
| | *N* (%) | *N* (%) | |
| **MATERNAL AUTONOMY INDEX** | | | **<0.001** |
| Low autonomy | 945 (21.1) | 698 (24.3) | |
| Moderate autonomy | 2183 (48.9) | 1413 (49.0) | |
| High autonomy | 1339 (30.0) | 770 (26.7) | |
| **CHILD FACTORS** | | | |
| **Age (months)** | | | **<0.001** |
| 0-11 | 1459 (32.7) | 416 (14.4) | |
| 12-23 | 1087 (24.3) | 909 (31.6) | |
| 24-35 | 827 (18.5) | 778 (27.0) | |
| 36-47 | 661 (14.8) | 513 (17.8) | |
| 48-59 | 433 (9.7) | 265 (9.2) | |
| **Age (months)** Mean (SD) | 22.9 (16.1) | 26.3 (13.9) | |
| **Sex,** Girl | 2406 (53.9) | 1355 (47.0) | **<0.001** |
| **Child had diarrhea recently,** Yes[††] | 965 (21.6) | 655 (22.8) | 0.104 |
| **MATERNAL FACTORS** | | | |
| **Age (years)** | | | **0.003** |
| 15-19 | 374 (8.4) | 187 (6.5) | |
| 20-24 | 1178 (26.5) | 853 (29.6) | |
| 25-29 | 1206 (27.0) | 758 (26.3) | |
| 30-34 | 859 (19.2) | 504 (17.4) | |
| 35-39 | 548 (12.4) | 348 (12.1) | |
| 40-44 | 220 (4.9) | 155 (5.4) | |
| 45-49 | 83 (1.6) | 77 (2.7) | |
| **Age (years)** Mean (SD) | 28.1 (6.8) | 28.3 (7.1) | |
| **Marital status** | | | 0.310 |
| Not married | 136 (3.0) | 67 (2.3) | |
| Married/cohabiting | 3928 (88.0) | 2547 (88.4) | |
| Divorced/separated/widowed | 404 (9.0) | 267 (9.3) | |
| **Number of other wives**[††] | | | 0.160 |
| 0 | 3472 (89.0) | 2196 (86.8) | |
| 1 | 390 (10.0) | 314 (12.4) | |
| 2+ | 44 (1.0) | 20 (0.8) | |
| **Mother's BMI**[††] | | | **<0.001** |
| Underweight (<18.5 kg/m$^2$) | 204 (4.6) | 204 (7.1) | |
| Normal (18.5-24.9 kg/m$^2$) | 3220 (72.7) | 2230 (77.8) | |
| Overweight (≥25.0 kg/m$^2$) | 1005 (22.7) | 434 (15.1) | |
| **Mother's education level** | | | **<0.001** |
| No education | 546 (12.3) | 481 (16.7) | |
| Primary | 2919 (65.3) | 1956 (67.9) | |
| Secondary or higher | 1002 (22.4) | 444 (15.4) | |
| **Occupation** | | | 0.050 |
| Housekeeping/agriculture | 3170 (71.0) | 2121 (73.6) | |
| Professional/service/manual labor | 1297 (29.0) | 760 (26.4) | |
| **Breastfeeding duration**[††] | | | **<0.001** |
| Never breastfed | 54 (1.2) | 38 (1.3) | |
| Stopped breastfeeding | 1923 (43.1) | 1442 (50.2) | |
| Still breastfeeding | 2482 (55.7) | 1393 (48.5) | |
| **Current pregnancy status,** Pregnant | 321 (7.2) | 317 (11.0) | **<0.001** |
| **HOUSEHOLD FACTORS** | | | |
| **Family wealth index** | | | **<0.001** |
| Poorest | 805 (18.0) | 675 (23.4) | |
| Poorer | 928 (20.8) | 719 (25.0) | |
| Middle | 899 (20.2) | 575 (20.0) | |
| Richer | 859 (19.2) | 523 (18.1) | |
| Richest | 976 (21.8) | 388 (13.5) | |
| **Number of HH members** | | | 0.774 |

| | | | |
|---|---|---|---|
| **Supplementary Table S1.** *Continued.* | | | |
| 1-5 | 2405 (53.8) | 1614 (56.0) | |
| 6-10 | 1944 (43.6) | 1200 (51.7) | |
| 11+ | 118 (2.6) | 67 (2.3) | |
| **Number of children under the age of 5** †† | | | 0.281 |
| 1 | 2386 (53.6) | 1499 (52.5) | |
| 2 | 1715 (38.5) | 1129 (39.5) | |
| 3+ | 351 (7.9) | 227 (8.0) | |
| **Type of area of residence,** Rural | 3690 (82.6) | 2546 (88.4) | **<0.001** |
| **Region** | | | 0.059 |
| Northern | 484 (10.8) | 319 (11.1) | |
| Central | 1958 (43.8) | 1306 (45.3) | |
| Southern | 2025 (45.4) | 1256 (43.6) | |
| **COMMUNITY FACTORS (Community Health Services)** | | | |
| **Antenatal attendance** | | | 0.355 |
| Never attended | 56 (1.3) | 51 (1.9) | |
| <4 visits (inadequate) | 2167 (48.5) | 1436 (49.7) | |
| ≥4 visits (recommended) | 2244 (50.2) | 1394 (48.4) | |
| **IFA supplementation,** Yes | 4061 (90.9) | 2566 (89.1) | **0.033** |
| **Received anthelmintic drugs during previous pregnancy,** Yes | 1932 (43.3) | 1148 (39.8) | **0.001** |

**Supplementary Table S1.** Chi-squared associations between demographic characteristics and child stunting ($N$ = 7,348). $p$, Pearson's chi-square $p$-value; †† Total count is less than $n$; BMI, body mass index; HH, household; IFA, iron and folic acid; SD, standard deviation. Statistical significance was set at $p < 0.05$. Significant $p$-values are in boldface.

| **Characteristics** | **Child stunting** |
|---|---|
| | *p-value* |
| Child's age (months) | 0.604 |
| Sex | 0.898 |
| Mother's age (years) | 0.119 |
| Mother's BMI | 0.909 |
| Mother's education level | 0.217 |
| Breastfeeding duration | 0.899 |
| Current pregnancy status | 0.128 |
| Family wealth index | 0.077 |
| Type of area of residence | 0.320 |
| IFA supplementation | 0.077 |
| Received anthelmintic drugs during previous pregnancy | 0.206 |

**Supplementary Table S2.** Interactions of maternal autonomy with other independent variables in predicting child stunting in Malawi ($N$ = 7,348). BMI, body mass index; IFA, iron and folic acid. Statistical significance was set at $p < 0.05$.

| Method | Child stunting |
|---|---|
| | AUC [95% CI] |
| Naïve | 0.65 [0.64, 0.67] |
| 10-fold cross-validated (IPCW) | 0.62 [0.60, 0.64] |
| 10-fold cross-validated (uncensored outcome) | 0.62 [0.60, 0.63] |
| cvAUROC (Mean ± SD) | 0.62 ± 0.03 |

**Supplementary Table S3.** *K*-fold cross-validation for estimating the accuracy of the predictive model for testing the association between maternal autonomy and child stunting in Malawi ($N$ = 7,348). Notes: Table shows the AUC values for the naïve and cvAUROC-based 10-fold cross-validated methods. cvAUROC = command for implementing *k*-fold cross-validation for the AUC for a binary outcome after fitting a logit or probit regression model in STATA. AUC, area under the curve; CI, confidence interval; IPCW, inverse probability of censoring weights; SD, standard deviation. Independent variables included all those analyzed in the GLMM-fitted regression analysis.

| Characteristics | MDHS 2010-2015/16 | | MDHS 2010 | | MDHS 2015/16 | |
|---|---|---|---|---|---|---|
| | **Full Dataset** ***n*=37092** | **Subset** ***n*=7348** | **Full Dataset** ***n*=19697** | **Subset** ***n*=3255** | **Full Dataset** ***n*=17395** | **Subset** ***n*=4093** |
| | ***N* (%)** | ***N* (%)** | ***N* (%)** | ***N* (%)** | ***N* (%)** | ***N* (%)** |
| **CHILD STUNTING** ‡ | 4030 (41.5) | 2881 (39.2) | 2144 (47.0) [1] | 1466 (45.0) | 1887 (36.6) [a] | 1415 (34.6) |
| **MATERNAL AUTONOMY** | | | | | | |
| **Maternal autonomy index** | | | | | | |
| Low autonomy | 8643 (23.3) | 1643 (22.4) | 4396 (22.3) | 688 (21.1) | 4247 (24.4) | 955 (23.3) |
| Moderate autonomy | 19310 (52.1) | 3596 (48.9) | 12136 (61.6) | 2004 (61.6) | 7174 (41.2) | 1592 (38.9) |
| High autonomy | 9139 (24.6) | 2109 (28.7) | 3165 (16.1) | 563 (17.3) | 5974 (34.4) | 1546 (37.8) |
| **CHILD FACTORS** | | | | | | |
| **Age (months),** Mean (SD) | 26.9 (16.6) | 27.6 (16.7) | 28.4 (6.8) | 23.1 (14.6) | 29.0 (17.2) | 25.1 (15.9) |
| 0-11 | 7477 (20.2) | 1875 (25.5) | 3965 (20.1) | 848 (26.0) | 3512 (20.2) | 1027 (25.0) |
| 12-23 | 7452 (20.1) | 1996 (27.2) | 4071 (20.7) | 998 (30.7) | 3382 (19.4) | 998 (24.4) |
| 24-35 | 7467 (20.1) | 1605 (21.8) | 4018 (20.4) | 726 (22.3) | 3449 (19.8) | 879 (21.5) |
| 36-47 | 7396 (19.9) | 1174 (16.0) | 3853 (19.6) | 426 (13.1) | 3543 (20.4) | 748 (18.3) |
| 48-59 | 7300 (19.7) | 698 (9.5) | 3791 (19.2) | 257 (7.9) | 3509 (20.2) | 441 (10.8) |
| **Sex,** Girl | 18562 (50.0) | 3761 (51.2) | 9889 (50.2) | 1660 (51.0) | 8674 (49.9) | 2101 (51.3) |
| **Child had diarrhea recently,** Yes φ | 6741 (19.7) | 1620 (22.1) | 3158 (17.7) [2] | 612 (18.8) | 3584 (22.0) [b] | 1008 (24.7) |
| **MATERNAL FACTORS** | | | | | | |
| **Age (years),** Mean (SD) | 28.3 (6.9) | 28.1 (6.8) | 28.7 (17.0) | 28.4 (7.0) | 28.1 (6.9) | 27.9 (6.9) |
| 15-19 | 2383 (6.4) | 561 (7.6) | 1126 (5.7) | 225 (6.9) | 1256 (7.2) | 336 (8.20) |
| 20-24 | 10755 (29.0) | 2031 (27.6) | 5567 (28.3) | 843 (25.9) | 5188 (29.8) | 1188 (29.0) |
| 25-29 | 9839 (26.5) | 1964 (26.7) | 5596 (28.4) | 975 (29.9) | 4243 (24.4) | 989 (24.2) |
| 30-34 | 6975 (18.8) | 1364 (18.6) | 3584 (18.2) | 548 (16.8) | 3391 (19.5) | 816 (19.9) |
| 35-39 | 4491 (12.1) | 895 (12.2) | 3256 (12.0) | 416 (12.8) | 2135 (12.3) | 479 (11.7) |
| 40-44 | 1919 (5.2) | 376 (5.1) | 1054 (5.3) | 171 (5.2) | 865 (5.0) | 205 (5.0) |
| 45-49 | 730 (2.0) | 160 (2.2) | 413 (2.1) | 78 (2.5) | 317 (1.8) | 82 (2.0) |
| **Marital status** | | | | | | |
| Not married | 898 (2.4) | 203 (2.8) | 330 (1.7) | 55 (1.7) | 568 (3.3) | 147 (3.6) |
| Married/cohabiting | 31720 (81.5) | 6475 (88.1) | 17133 (87.0) | 3032 (93.1) | 14587 (83.9) | 3443 (84.1) |
| Divorced/separated/widowed | 4474 (12.1) | 670 (9.1) | 2234 (11.3) | 168 (5.2) | 2240 (12.8) | 503 (12.3) |
| **Number of other wives** †† | | | | | | |
| 0 | 27282 (86.6) | 5668 (88.1) | 14613 (86.0) [2] | 2640 (87.8) [2] | 12669 (87.3) [b] | 3028 (88.3) [b] |
| 1 | 3850 (12.2) | 704 (10.9) | 2176 (12.8) [2] | 337 (11.2) [2] | 1674 (11.5) [b] | 367 (10.7) [b] |
| 2+ | 386 (1.2) | 64 (1.0) | 210 (1.2) [2] | 30 (1.0) [2] | 176 (1.2) [b] | 34 (1.0) [b] |
| **Mother's BMI (kg/m$^2$)** †† φ | | | | | | |
| Underweight (<18.5) | 720 (5.7) | 408 (5.6) | 423 (6.3) [2] | 196 (6.1) | 298 (5.0) [b] | 212 (5.3) |
| Normal (18.5-24.9) | 9362 (74.1) | 5449 (74.7) | 5043 (74.6) [2] | 2456 (75.9) | 4319 (73.5) [b] | 2993 (73.7) |

**Supplementary Table S4.** *Continued.*

| | | | | | | | | |
|---|---|---|---|---|---|---|---|---|
| Overweight (≥25.0) | 2555 (20.2) | 1439 (19.7) | | 1293 (19.1) [2] | 585 (18.0) | | 1261 (21.5) [b] | 854 (20.0) |
| **Mother's education level** | | | | | | | | |
| No education | 5772 (15.6) | 1028 (14.0) | | 3441 (17.5) | 513 (15.8) | | 2331 (13.4) | 515 (12.6) |
| Primary | 24894 (67.1) | 4874 (66.3) | | 13345 (67.7) | 2205 (67.8) | | 11549 (66.4) | 2669 (65.2) |
| Secondary or higher | 6426 (17.3) | 1446 (19.7) | | 2911 (14.8) | 537 (16.4) | | 3515 (20.2) | 909 (22.2) |
| **Occupation** | | | | | | | | |
| Housekeeping/agriculture | 26714 (72.0) | 5291 (72.0) | | 13835 (70.2) | 2276 (70.0) | | 12880 (74.0) | 3015 (73.7) |
| Professional/service/manual labor | 10378 (28.0) | 2057 (28.0) | | 5862 (29.8) | 979 (30.0) | | 4515 (26.0) | 1078 (26.3) |
| **Breastfeeding duration** [†† φ] | | | | | | | | |
| Never breastfed | 903 (2.5) | 92 (1.3) | | 425 (2.2) [2] | 24 (0.7) | | 478 (2.8) | 68 (1.7) |
| Stopped breastfeeding | 21933 (59.8) | 3364 (45.9) | | 11246 (58.3) [2] | 1290 (39.8) | | 10687 (61.4) | 2075 (50.7) |
| Still breastfeeding | 13842 (37.7) | 3875 (52.8) | | 7611 (39.5) [2] | 1925 (59.5) | | 6230 (35.8) | 1950 (47.6) |
| **Current pregnancy status,** Pregnant | 2984 (8.1) | 638 (8.7) | | 1814 (9.2) | 331 (10.2) | | 1170 (6.7) | 308 (7.5) |
| **HOUSEHOLD FACTORS** | | | | | | | | |
| **Household wealth index** | | | | | | | | |
| Poorest | 8546 (23.0) | 1480 (20.2) | | 4252 (21.6) | 541 (16.6) | | 4293 (24.7) | 939 (23.0) |
| Poorer | 8224 (22.2) | 1647 (22.4) | | 4307 (21.9) | 716 (22.0) | | 3918 (22.5) | 931 (22.7) |
| Middle | 7641 (20.6) | 1474 (20.0) | | 4276 (21.7) | 689 (21.2) | | 3364 (19.3) | 785 (19.1) |
| Richer | 6675 (18.0) | 1383 (18.8) | | 3650 (18.5) | 639 (19.6) | | 3025 (17.4) | 744 (18.2) |
| Richest | 6006 (16.2) | 1364 (18.6) | | 3211 (16.3) | 670 (20.6) | | 2795 (16.1) | 694 (17.0) |
| **Number of HH members** | | | | | | | | |
| 1-5 | 20040 (54.0) | 4019 (54.7) | | 10233 (52.0) | 1605 (49.3) | | 9807 (56.4) | 2414 (59.0) |
| 6-10 | 16092 (43.4) | 3144 (42.8) | | 8858 (45.9) | 1531 (47.0) | | 7234 (41.6) | 1614 (39.4) |
| 11+ | 960 (2.6) | 185 (2.5) | | 606 (3.1) | 119 (3.7) | | 354 (2.0) | 65(1.6) |
| **Number of children under the age of 5** [††] | | | | | | | | |
| 1 | 15168 (42.4) | 3885 (53.2) | | 7017 (37.0) [2] | 1485 (45.9) [2] | | 8151 (48.5) [b] | 2400 (58.9) [b] |
| 2 | 16404 (45.9) | 2844 (38.9) | | 9272 (49.0) [2] | 1415 (43.8) [2] | | 7132 (42.4) [b] | 1429 (35.1) [b] |
| 3+ | 4191 (11.7) | 578 (7.9) | | 2652 (14.0) [2] | 332 (10.2) [2] | | 1539 (9.1) [b] | 246 (6.0) [b] |
| **Type of area of residence,** Rural | 31956 (86.2) | 6237 (84.9) | | 166878 (85.7) | 2718 (83.5) | | 15077 (86.7) | 3519 (86.0) |
| **Region** | | | | | | | | |
| Northern | 4282 (11.6) | 804 (10.9) | | 2310 (11.7) | 344 (10.5) | | 1972 (11.3) | 460 (11.2) |
| Central | 15852 (42.7) | 3264 (44.4) | | 8449 (42.9) | 1496 (46.0) | | 7403 (42.6) | 1768 (43.2) |
| Southern | 16958 (45.7) | 3281 (44.7) | | 8938 (45.4) | 1415 (43.5) | | 8021 (46.1) | 1865 (45.6) |
| **COMMUNITY FACTORS** | | | | | | | | |
| **Antenatal attendence** [φ] | | | | | | | | |

**Supplementary Table S4.** *Continued.*

| | | | | | | | | |
|---|---|---|---|---|---|---|---|---|
| Never attended | 460 (1.7) | 107 (1.5) | | 215 (1.6) [2] | 34 (1.0) | | 245 (1.8) [b] | 73 (1.8) |
| <4 visits (inadequate) | 13508 (49.7) | 3606 (49.0) | | 7126 (52.2) [2] | 1695 (52.1) | | 6382 (47.2) [b] | 1908 (46.6) |
| ≥4 visits (recommended) | 13211 (48.6) | 3639 (49.5) | | 6323 (46.2) [2] | 1526 (46.9) | | 6888 (51.0) [b] | 2112 (51.6) |
| **IFA supplementation,** Yes [φ] | 24541 (90.3) | 6627 (90.2) | | 12464 (91.2) [2] | 2962 (91.0) | | 12077 (89.4) [b] | 3665 (89.5) |
| **Received anthelmintic drugs during previous pregnancy,** Yes [φ] | 10713 (39.4) | 3080 (41.9) | | 3743 (27.4) [2] | 933 (28.7) | | 6970 (51.6) [b] | 2147 (52.5) |

**Supplementary Table S4.** Comparison of participants' characteristics in the full and subset data in each survey year. [‡] *n* = 9,717; [φ] Total count is less than *n* in pooled data (full dataset); [††] Total count is less than *n* in pooled data (MDHS 2010–2015/16 subset and full dataset) and individual MDHS 2010 and 2015/16 subset data, respectively; [1] *n* = 4,558; [2] total of variable category counts is less than *n* in MDHS 2010 data; [a] *n* = 5,159; [b] total of variable category counts is less than *n* in MDHS 2015/16 data; BMI, body mass index; IFA, iron and folic acid; MDHS, Malawi Demographic and Health Survey; SD, standard deviation.

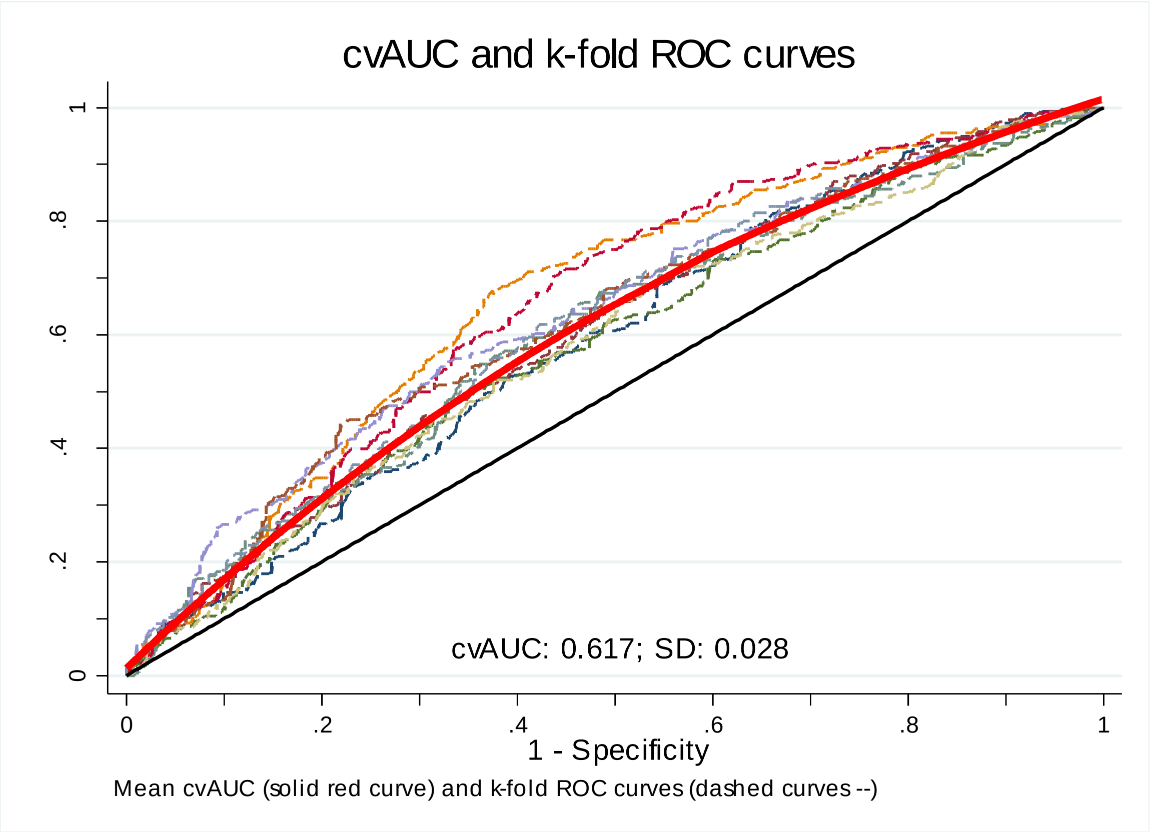


**Supplementary Figure S1.** *K*-fold cross-validation for estimating the accuracy of the predictive model for testing the association between maternal autonomy and child stunting in Malawi.
